# RNA thermosensors facilitate *Streptococcus pneumoniae* and *Haemophilus influenzae* immune evasion

**DOI:** 10.1371/journal.ppat.1009513

**Published:** 2021-04-29

**Authors:** Hannes Eichner, Jens Karlsson, Laura Spelmink, Anuj Pathak, Lok-To Sham, Birgitta Henriques-Normark, Edmund Loh

**Affiliations:** 1 Department of Microbiology, Tumor and Cell Biology, Karolinska Institutet, Solna, Sweden; 2 Infectious Disease Programme, Yong Loo Lin School of Medicine, National University of Singapore, Singapore, Singapore; 3 Department of Microbiology and Immunology, Yong Loo Lin School of Medicine, National University of Singapore, Singapore, Singapore; 4 Clinical Microbiology, Bioclinicum, Karolinska University Hospital, Solna, Sweden; 5 Lee Kong Chian School of Medicine and Singapore Centre on Environmental Life Sciences Engineering, Nanyang Technological University, Singapore, Singapore; University of Birmingham, UNITED KINGDOM

## Abstract

Bacterial meningitis is a major cause of death and disability in children worldwide. Two human restricted respiratory pathogens, *Streptococcus pneumoniae* and *Haemophilus influenzae*, are the major causative agents of bacterial meningitis, attributing to 200,000 deaths annually. These pathogens are often part of the nasopharyngeal microflora of healthy carriers. However, what factors elicit them to disseminate and cause invasive diseases, remain unknown. Elevated temperature and fever are hallmarks of inflammation triggered by infections and can act as warning signals to pathogens. Here, we investigate whether these respiratory pathogens can sense environmental temperature to evade host complement-mediated killing. We show that productions of two vital virulence factors and vaccine components, the polysaccharide capsules and factor H binding proteins, are temperature dependent, thus influencing serum/opsonophagocytic killing of the bacteria. We identify and characterise four novel RNA thermosensors in *S*. *pneumoniae* and *H*. *influenzae*, responsible for capsular biosynthesis and production of factor H binding proteins. Our data suggest that these bacteria might have independently co-evolved thermosensing abilities with different RNA sequences but distinct secondary structures to evade the immune system.

## Author summary

*Streptococcus pneumoniae* and *Haemophilus influenzae* are bacteria that reside in the upper respiratory tract. This harmless colonisation can progress to severe and often lethal septicaemia and meningitis. However, the molecular mechanisms governing the transition from carriage to disease remain largely unknown. Our work here shows that both *S*. *pneumoniae* and *H*. *influenzae* evade defensive mechanisms of the innate immune system by sensing body temperature. We identified four novel RNA thermosensors that influence production of the protective capsules, as well as immune modulatory Factor H binding proteins. These RNA thermosensors are located in the beginning of the protein-coding mRNAs and modulate the production of these virulence factors depending on temperature. The body core temperature is slightly higher than on the surface of the upper respiratory tract. Here we describe RNA thermosensors that are able to sense these changes and activate stronger protection against the immune system. Capsule and Factor H binding protein are important vaccine components and temperature regulation might impact their efficiency. Identification of such regulatory RNAs enables clinicians, microbiologists and public health practitioners to adjust their diagnostic techniques, and treatments to best fit the condition of the patients.

## Introduction

*Streptococcus pneumoniae* and *Haemophilus influenzae* are human restricted respiratory pathogens, often found colonising the nasopharynx, capable of causing deadly meningitis, pneumonia and sepsis. In order to survive in the host, both bacteria have evolved analogous survival mechanisms, such as production of polysaccharide capsules to evade host immune responses. Encapsulated bacterial pathogens pose a major threat to human health and contribute to significant morbidity and mortality [1]. For Gram-positive pneumococci, the capsule enables the bacteria to avoid phagocytosis, with 100 different capsular serotypes identified as of May 2020 [2]. Pneumococci have also been suggested to be resistant to complement killing due to their capsule [3]. The Gram-negative *H*. *influenzae* shows six capsular serotypes, facilitating the bacterium to resist phagocytosis and complement-mediated killing [4]. Polysaccharide-based vaccines are used to prevent *S*. *pneumoniae* and *H*. *influenzae* infections [5].

In addition to capsules, these two pathogens could also evade complement-mediated killing by binding to human Factor H (FH), the major negative regulator of the alternative complement pathway. FH is recruited through high affinity interactions with FH binding proteins expressed on the bacterial surface, such as the Pneumococcal Surface Protein C (PspC) and the *H*. *influenzae* Protein H (PH) [6–8]. Currently, FH binding protein is a main component of two licensed vaccines (Bexsero and Trumenba) used against *Neisseria meningitidis*. Pneumococcal PspC has also been suggested as a potential vaccine candidate against pneumococcal infections [9–11].

The ecological niche for *S*. *pneumoniae* and *H*. *influenzae* is the nasopharyngeal cavity from where they may spread into the bloodstream and cause disease. There is a temperature gradient in the nasal cavity where these two pathogens colonise. The temperature on the surface of the anterior nares is around 30°C to 32°C at the end of inspiration, and rises to around 34°C in the posterior nasopharynx and tonsillar region [12,13]. Both these sites on the mucosal surface are significantly cooler than the core body temperature of 37°C, where the bacteria replicate during invasive diseases. Previously, we showed that temperature acts as a danger signal to *N*. *meningitidis*, prompting the bacterium to enhance expression of immune evasion factors via three independent RNA thermosensors (RNAT) [14]. RNATs are elements usually located in the 5´-untranslated region (5´-UTR) of an mRNA transcript, forming a secondary structure at lower temperature that inhibits protein translation by blocking access of ribosomes to the ribosome binding site (RBS). As temperature rises, the RNA secondary structure undergoes a conformational change due to higher thermodynamic energy, exposing the RBS thus allowing translation [15].

Recently, we demonstrated that specific variants of meningococcal capsular RNATs resulted in hypercapsulation and were associated with episodes of invasive meningococcal diseases [16]. Hence, the importance of temperature sensing in meningococci prompted us to investigate whether temperature could affect expression of virulence factors in other meningitis-causing pathogens that colonise the same niche, i.e. *S*. *pneumoniae* and *H*. *influenzae*. Although the role of the polysaccharide capsule and FH binding proteins in *S*. *pneumoniae* and *H*. *influenzae* in complement evasion has been studied, their regulatory mechanisms remain largely unknown.

Here, we identify that temperature plays an important role in governing the production of the capsular polysaccharide and FH binding proteins in *S*. *pneumoniae* and *H*. *influenzae*, thus facilitating complement-mediated evasion. Furthermore, through sequence analyses of the RNATs, we observed that the three meningitis-causing pathogens have unique RNA sequences, with no sequence conservation, but that they are able to serve the same thermosensing function.

## Results

**Fig 1:**
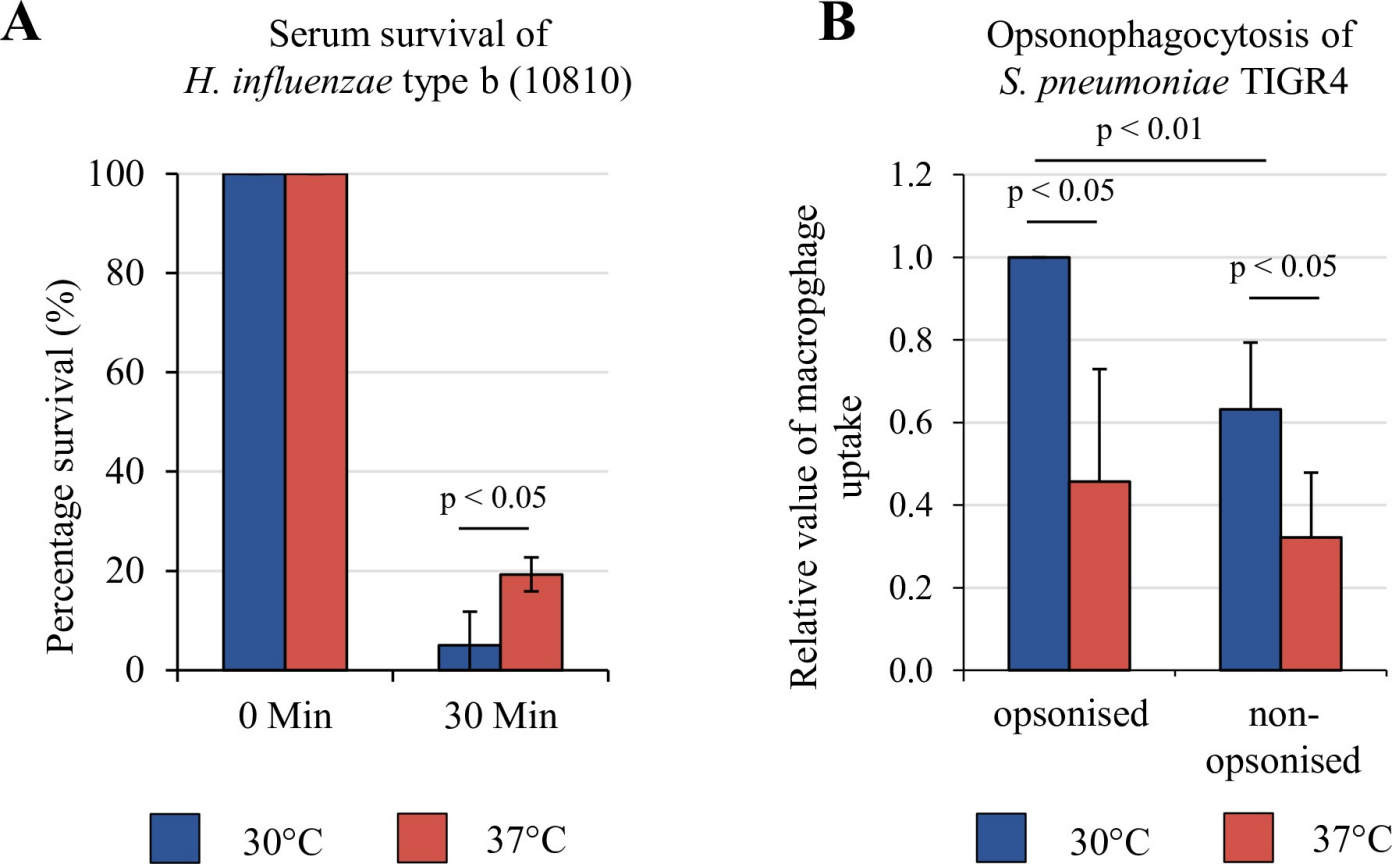
Temperature influences serum killing and opsonophagocytosis of *H*. *influenzae* and *S*. *pneumoniae*, respectively. (A) Serum killing of *H*. *influenzae* type b was measured by pre-incubating the bacteria at 30°C or 37°C in RMPI for 1 hour prior to addition of 25% pooled human serum. The mixtures were subjected to serum killing at 37°C for 30 minutes. *H*. *influenzae* type b pre-incubated at 37°C shows higher resistance to complement-mediated killing than bacteria at 30°C. (B) The effect of temperature on opsonophagocytosis of pneumococci by THP-1 macrophages was analysed by growing *S*. *pneumoniae* TIGR4 bacteria at either 30°C or 37°C. The bacteria were then opsonised with pooled human serum for 30 minutes at 37°C. Non-opsonised bacteria were exposed to only RPMI for 30min at 37°C. Pneumococci grown at 37°C are more resistant to phagocytosis by macrophages than bacteria grown at 30°C. Error bars denote s.e.m. Statistical significance calculated using a paired, two-tailed student t-test.

When *S*. *pneumoniae* (TIGR4, serotype 4) and *H*. *influenzae* type b (Hib, strain 10810) were grown in liquid (C + Y and supplemented BHI respectively) at temperature ranging from 30°C to 40°C, no major growth impairments were observed. Both bacterial strains also showed similar growth when incubated at 30°C up to 42°C on agar plates. To investigate whether temperature could alter the bacterial survival or uptake ability by host cells, we performed serum killing and opsonophagocytosis using bacteria incubated at different temperature. Similar to the method used for meningococci [14], *H*. *influenzae* type b was grown overnight at 30°C, harvested and pre-incubated at 30°C or 37°C for one hour prior to 25% human serum killing assays. *H*. *influenzae* pre-incubated at 37°C showed a significantly higher serum survival rate after 30 minutes compared to those at 30°C (Fig 1A). Since pneumococci are naturally resistant to serum killing, the effect of different temperature was investigated using opsonophagocytosis assays. *S*. *pneumoniae* was grown at either 30°C or 37°C and opsonised with human serum prior to challenge of human THP-1 macrophages. Serum opsonised *S*. *pneumoniae* grown at 37°C was phagocytosed significantly less than those grown at 30°C (Fig 1B). Our results suggest that both *S*. *pneumoniae* and *H*. *influenzae* are able to evade complement-mediated killing in a temperature dependent manner.

**Fig 2:**
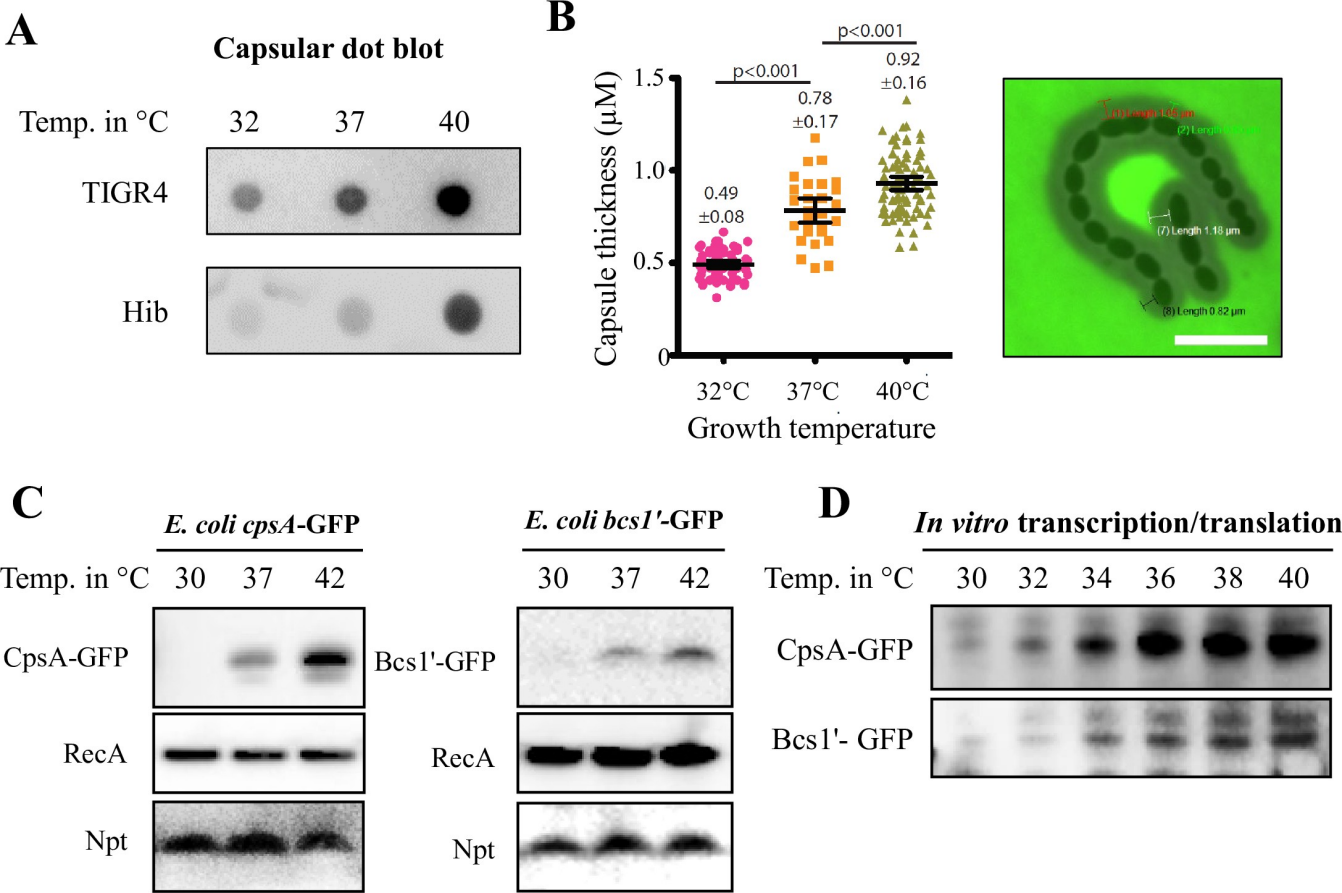
Capsular gene expression and production of capsules of *S*. *pneumoniae* and *H*. *influenzae* are temperature regulated. (A) Capsular dot blot analyses of *S*. *pneumoniae* and *H*. *influenzae* type b (Hib) show increased presence of capsular components in culture supernatants with increasing temperature. Pneumococcal Serotype 4 and *H*. *influenzae* type b polysaccharide capsule specific anti-sera were used for detection of capsules. (B) The *S*. *pneumoniae* capsule is thicker at higher temperature as shown by dextran exclusion assay (right panel, bar = 5μm). The average thickness of pneumococcal capsules (μm) grown at different temperature were tabulated and shown in the graph with standard deviations (+/-). (C) Thermoregulation of CpsA and Bcs1´ UTR-*gfp* fusion products detected in *E*. *coli* by Western blot analysis (Anti-GFP antibody used for detection of GFP, RecA and Neomycin-phosphotransferase used as controls). (D) Western blots of *in vitro* transcription/translation assay of CpsA and Bcs1´ UTR-*gfp* fusion products show temperature regulation of CpsA-GFP and Bcs1´-GFP. (Anti-GFP antibody used for detection of GFP). Error bars denote s.e.m. Statistical significance calculated using a paired, two-tailed student t-test.

The amount of capsular polysaccharide has been shown to impact phagocytosis by host cells. Therefore, we analysed the production of capsular polysaccharides of *S*. *pneumoniae* grown at different temperature by performing dot blot assay [17]. Dot blots using serotype 4 specific anti-serum showed that the polysaccharide capsule was produced in higher amounts with increasing temperature in the bacterial supernatant (Fig 2A). This is in agreement with previous studies, showing that capsular shedding enable pneumococci to evade complement killing and facilitate bacterial spread [18,19]. These shed capsules are immunogenic and free-floating components that could additionally sequester antibodies and immune cells. To further confirm our findings as well as investigating whether this temperature dependent phenotype is pneumococcal serotype independent, we visualised and quantified the capsular expression by performing a FITC-dextran exclusion assay using the *S*. *pneumoniae* strain NUS0276 (derived from *S*. *pneumoniae* strain D39 (serotype 2) expressing the serotype 19F capsular locus). Indeed, we observed that the polysaccharide thickness increased with temperature also in strain NUS0276 (Fig 2B). Likewise, for *H*. *influenzae*, a dot blot assay was performed using a *H*. *influenzae* serotype B specific anti-sera. Results also showed higher presence of capsular polysaccharides in the culture supernatant at higher temperature (Fig 2A). We conclude that capsular expression in pneumococci and *H*. *influenzae* increases with temperature.

Next, to investigate if the polysaccharide capsular gene operons of *S*. *pneumoniae* and *H*. *influenzae* harbour putative RNATs, we examined the intergenic regions upstream of their respective gene loci. The capsular operon promoter of the pneumococcal strain TIGR4 has previously been described and is located 30 bps upstream of the *cpsA* start codon with a short 5´-UTR (26 nts) [17]. In *H*. *influenzae* type b 10810, we identified a putative σ-70 promoter, 215 bps upstream of the capsular biosynthesis operon (*bcs1´*) using the promoter prediction programme BPROM (Softberry Inc., Mt. Kisco, NY) [20]. Using Vienna *RNAfold* package [21] and *VARNA* applet [22], we determined the putative secondary structures of *cpsA* and *bcs1´* 5´-UTRs together with their first 24-coding bases. Consistent with the classical feature of an RNAT, both *cpsA* and *bcs1’* 5´- UTR regions form stem-loop structures that occlude the RBS (S1A and S1B Fig).

RNATs are elements located within specific 5´-UTR of mRNAs and they could function in a heterologous host [14,23]. Therefore, the whole intergenic regions upstream of *cpsA* and *bcs1’* together with their respective first 24 coding-bases were translationally fused to a green fluorescent protein (GFP) within the plasmid pEGFP-N2 and transformed into *Escherichia coli*. Thermoregulation of CpsA-GFP and Bcs1’-GFP was evident in *E*. *coli* (Fig 2C). Anti-recombinase A (RecA) antibody (Abcam) was used as a cytoplasmic loading control and anti-Neomycin-Phosphotransferase (Npt) antibody to eliminate potential effects of temperature on plasmid replication (Fig 2C). The two previously identified RNA thermosensors, the neisserial capsular CssA and the listerial transcriptional activator PrfA, were used as positive controls (S1C Fig)

To eliminate temperature dependent transcription, the expression of *cpsA* and *bcs1’* mRNAs were examined by Northern blotting. Both mRNA transcript levels were unaffected by temperature (S2 Fig), indicating that upregulation of CpsA and Bcs1’ occurs post-transcriptionally. To further eliminate the involvement of host dependent factors in their thermoregulation, we subjected these GFP-fusion constructs to an *in vitro* transcription/translation assay at different biological relevant temperature ranging from 30°C to 40°C (Fig 2D). Both CpsA and Bcs1’ thermosensors displayed gradual increment of expression, similar to the rheostat expression of the meningococcal CssA (S1D Fig) [14]. In contrast, the PrfA RNAT of the enteric pathogen *L*. *monocytogenes* displayed an on-off expression switch at 36–38°C (S1D Fig). These results confirm the presence of *bona fide* RNATs controlling the capsular biosynthesis operons of *S*. *pneumoniae* and *H*. *influenzae*.

**Fig 3:**
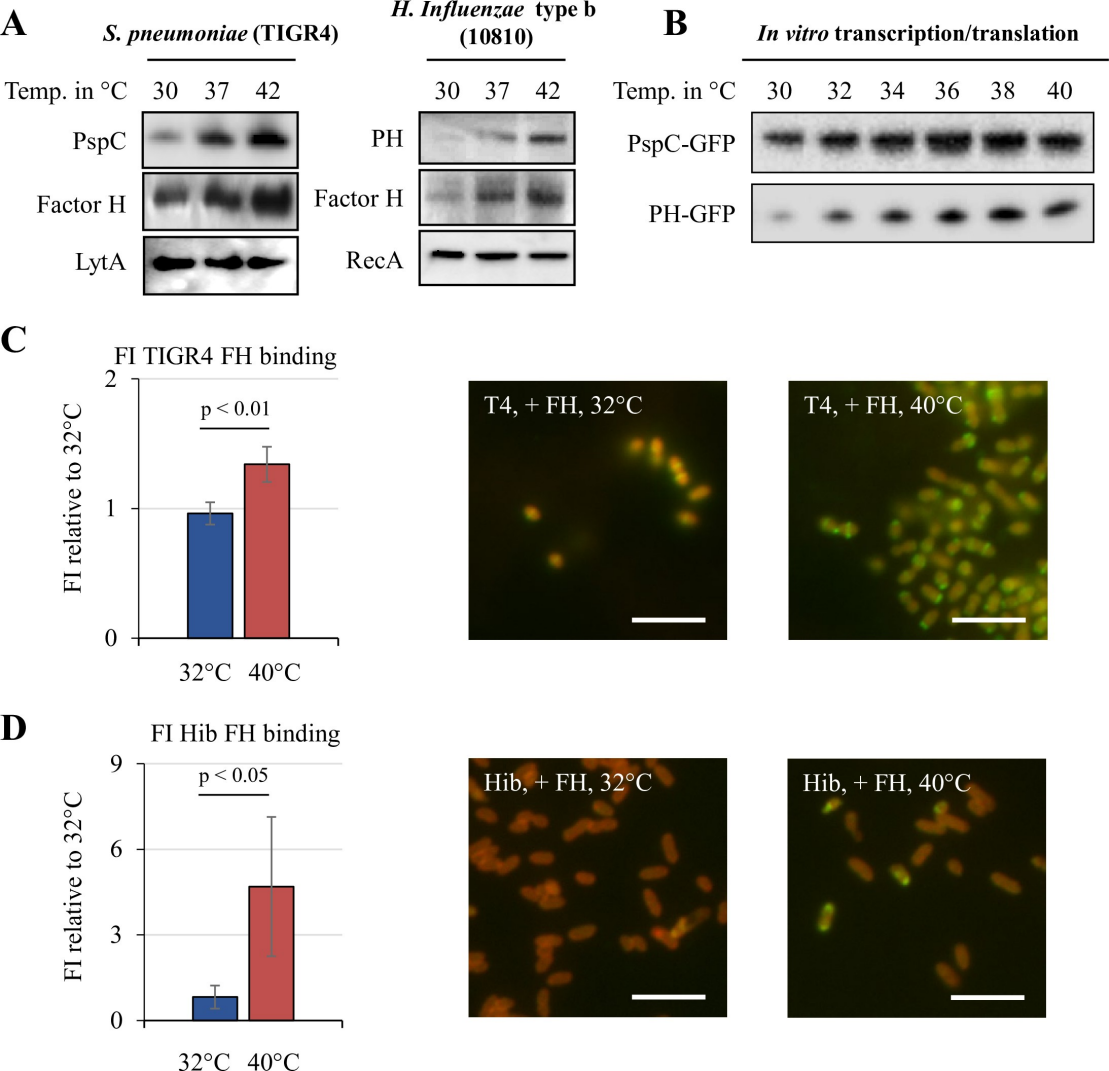
Factor H (FH) binding protein expression of *S*. *pneumoniae* and *H*. *influenzae* is temperature regulated. (A) Western and far-western blot analyses show thermoregulated PspC and FH expression in *S*. *pneumoniae*. Anti-PspC (*S*. *pneumoniae*) and anti-PH (*H*. *influenzae*) antibodies used for detection of respective FH binding protein. Human FH was used to determine binding to respective FH binding protein. Anti-human FH antibody was used. LytA (*S*. *pneumoniae*) and RecA (*H*. *influenzae*) were used as loading controls. (B) Western blots of *in vitro* transcription/translation assay of PspC and PH UTR-*gfp* fusion products show temperature regulation of PspC-GFP and PH-GFP. (Anti-GFP antibody used for detection of GFP). (C) Fluorescent flow cytometry shows increased human FH binding to *S*. *pneumoniae* grown at higher temperature. Fluorescent microscopy analysis reveals that more FH is bound to *S*. *pneumoniae* at 40°C (FH binding in a banded pattern). (D) Fluorescent flow cytometry shows increased human FH binding to *H*. *influenzae* type b (Hib) grown at higher temperature. Fluorescent microscopy analysis reveals a FH positive population of *H*. *influenzae* type b along with a FH negative population at 40°C. (C & D) Fluorescence intensities of three experiments were pooled and FH binding of bacteria grown at 40°C vs 32°C analysed. Error bars denote s.e.m. Statistical significance calculated using a paired, two-tailed student t-test.

In addition to capsules, pneumococci and *H*. *influenzae* could also evade complement-mediated killing by binding to FH. Therefore, we examined whether the FH binding proteins PspC in *S*. *pneumoniae* and PH in *H*. *influenzae* might also be thermoregulated. Using strain-specific anti-Factor H binding protein antibodies, we detected an increased expression of PspC and PH at higher temperature. By performing far western blotting using purified human Factor H, we demonstrate more human FH binding with increasing temperature, consistent with the higher expression of PspC or PH (Fig 3A). Expression of *pspC* and *lph* mRNA transcripts was unaffected by temperature changes (S2 Fig), suggesting that thermoregulation also occurs post-transcriptionally, similar to their capsular gene counterparts. This finding prompted us to investigate the presence of RNAT structures within the 5´-region of pneumococcal *pspC* and the *H*. *influenzae lph* mRNAs. Using promoter prediction programs, we identified two σ-^70^ promoters; 65 and 64 bps upstream from the start codon of the *pspC* and *lph* operons, respectively. Next, we reviewed the putative secondary structures of *pspC* and *lph* using Vienna *RNAfold* package and identified putative RNAT secondary structures with occluded RBS (S2 Fig). Next, the whole intergenic regions upstream of *pspC* and *lph* together with their respective first 90 coding bps were cloned into GFP-expressing plasmids pEGFP-N2 and transformed into *E*. *coli*. To eliminate any bacterial host factor involvement, these GFP-fusion constructs were subjected to *in vitro* transcription/translation at different biological relevant temperature (Fig 3B). The PspC and PH thermosensors displayed gradual increment of expression, similar to the rheostat expression of their capsular counterparts CpsA and Bcs1’.

The finding of elevated FH binding proteins at higher temperature from protein lysates is not a definite evidence that more surface exposed proteins are produced, nor that FH binding occurs at the bacterial surface. To establish whether temperature could affect binding of FH, *S*. *pneumoniae* and *H*. *influenzae* grown at different temperature were exposed to fluorescent labelled FH and analysed by flow cytometry. *S*. *pneumoniae* showed an increased binding of FH when grown at 40°C, compared to 32°C. Fluorescence microscopy staining was consistent in showing higher intensity of FH binding in *S*. *pneumoniae* grown at 40°C compared to 32°C (Fig 3C). Similarly, *H*. *influenzae* grown at different temperature also showed differences in the flow cytometry analysis (Fig 3D). Two populations were observed when grown at 40°C as indicated by the two maxima in the histograph (S3 Fig). When exposed to labelled FH, bacteria from this culture showed a clear increase in fluorescence intensity. Fluorescence microscopy staining revealed almost no fluorescence on bacteria grown at 32°C. When grown at 40°C, a subpopulation was found that was strongly positive for FH, while remaining bacteria were negative. The microscopy observation is consistent with the two maxima in flow cytometry (Fig 3D)

**Fig 4:**
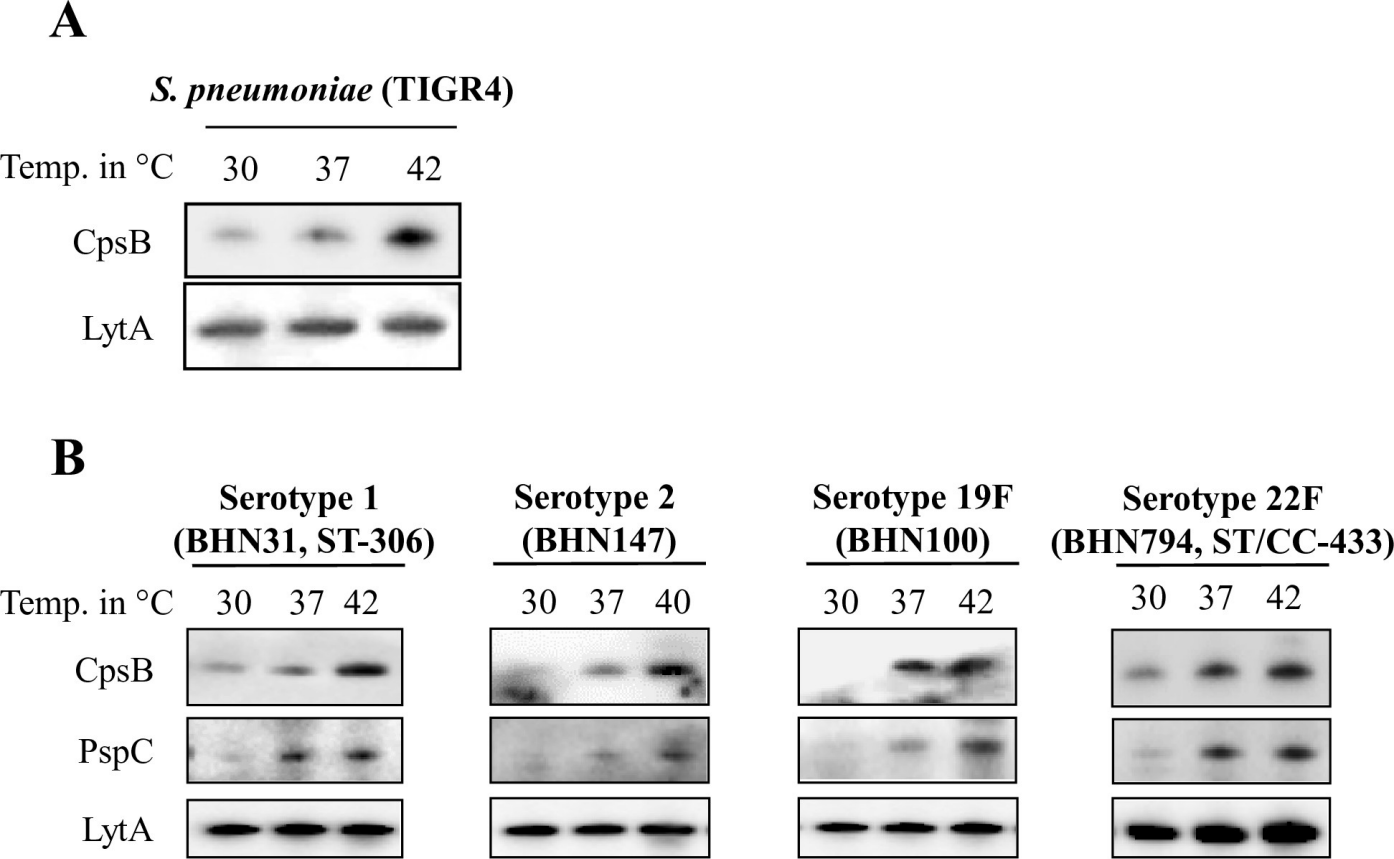
Clinical isolates of *S*. *pneumoniae* also possess thermosensing capability. (A) Thermoregulation of CpsB in *S*. *pneumoniae* TIGR4 as detected by western blot analysis. (B) Thermoregulation of CpsB and PspC, using western blot analyses, in clinical pneumococcal isolates of serotypes 1, 2 19F and 22F. LytA was used as a loading control for all panels.

Moreover, we were interested to see if this temperature sensing phenomenon in pneumococci is also present in clinical strains of different serotypes. Sequence conservation or polymorphism of the RNATs in different invasive strains could indicate clinical relevance as exemplified by the capsular RNAT of *N*. *meningitidis* [16]. The first four genes of the pneumococcal capsular locus (*cpsABCD*) are common to all serotypes and are controlled by a single promoter [24]. Due to unavailability of antibody against capsular polysaccharide synthesis protein A (CpsA), we investigated capsular synthesis proteins in *S*. *pneumoniae* TIGR4 using antibody against the second capsular polysaccharide synthesis protein (CpsB) [25]. The RNAT governing *cpsA* should also affect the expression of *cpsB* as the proteins are encoded on a polycistronic mRNA. Using *S*. *pneumoniae* TIGR4, we show that CpsB production is also temperature dependent (Fig 4A), suggesting that the pneumococcal capsular RNAT controls the downstream protein production and that CpsB levels could be used as a marker of pneumococcal capsule production. In addition to the laboratory strains TIGR4 (serotype 4) and D39 (serotype 2) (Fig 2B), we selected four clinical pneumococcal strains of serotypes 1, 2, 19F and 22F, and subjected them to different temperature growth. Thermoregulation of CpsB was also evident in these isolates (Fig 4B). Through genome comparison analyses, we identified that the nucleotide sequences within the 5´-UTR of *cpsA*, essential for forming the RNA secondary structure are highly conserved among these serotypes (S4 Fig). Natural occurring polymorphisms in the *cps* 5´-UTR of serotypes 1, 2, 19F and 22F could neither alter the putative RNA secondary structures, nor the thermoregulation ability of CpsB. Thermosensing of PspC was also observed in these isolates (Fig 4B). Genome comparison of the 5´-regions of the *pspC* mRNA show full conservation within the 5´-UTR, with polymorphisms occurring only within the coding mRNA (S5 Fig). Increased production of the PspC protein at higher growth temperature suggests that 5´-sequence conservation is favoured to maintain the thermosensing ability.

To investigate the sequence distribution of the RNATs within *S*. *pneumoniae* and *H*. *influenzae* genomes, we performed a nucleotide BLAST search for each RNAT in the database of the National Center for Biotechnology Information (NCBI) (Table 1). Our analysis revealed that the proposed RNAT sequences for *cpsA* and *pspC* are highly conserved in many sequenced strains of *S*. *pneumoniae*. We identified a few single nucleotide polymorphisms (SNPs) which are identical to those reported in S4 and S5 Figs. For *H*. *influenzae*, we only observed a few alignments for *bcs1´* and *lph* RNAT sequences, with all sharing high sequence identity.

**Table 1:** 5′-UTR sequences of *cpsA*, *pspC*, *bcs1´*, *lph* were analysed using nBLAST for occurrences in other isolates. Number of isolates identified (n).

Species	Genes	Isolates (n)	Identity range	Cause for nucleotide differences
*S*. *pneumoniae*	*cpsA*	278	93.75–100%	SNPs and incomplete coverage
	*pspC*	30	95.4–100%	SNPs and incomplete coverage
*H*. *influenzae*	*bcs1´*	13	95–100%	SNPs and incomplete coverage
	*lph*	4	76–100%	Incomplete coverage

## Discussion

*S*. *pneumoniae* and *H*. *influenzae* are major causative agents of bacterial meningitis in children and adults [26]. These pathogens have evolved to colonise the human nasopharynx, a region replete of other bacteria and environmental stress factors. In addition, acute inflammation and pyretic response caused by systemic diseases such as influenza viruses could alter the local temperature within the nasopharynx [27]. It is known that humans as well as mice harbouring an influenza A virus infection are highly susceptible to a secondary pneumococcal infection [28,29], but whether the fever response to the viral infection is a predisposing factor is currently unknown.

Temperature-mediated production of capsules and binding of human FH in *S*. *pneumoniae* and *H*. *influenzae* could provide better protection for the bacteria from immune killing. It has previously been reported that the promoter sequence of the capsular operon in the pneumococcus is important for its virulence [17]. Our results here suggest that a capsular RNAT could serve as an additional layer of capsule regulation as this RNAT is able to function outside of its natural environment in the absence of pneumococcal factors. It has previously been shown that high capsular expression in pneumococci may have negative effects on adhesion to the respiratory mucosa [30]. Additionally, the PneumoExpress platform, a transcriptomic study by Aprianto *et al*. 2018 shows no upregulation of *cps* transcript at higher temperature further strengthens our hypothesis that an RNAT is controlling the production of pneumococcal capsule at the translation initiation step [31]. Thermosensor-mediated modulation of capsular production may therefore be a central strategy for *S*. *pneumoniae* and *H*. *influenzae* to optimally colonise their normal habitat, the human nasopharynx. At the same time, and when necessary (i.e. local inflammation), these bacteria could rapidly express certain properties to avoid immune killing. Another important survival strategy for *S*. *pneumoniae* and *H*. *influenzae* is the ability to bind FH using their surface exposed Factor H binding proteins. However, overexpression of such factors at certain sites, such as on nasopharyngeal mucosal surfaces, could evoke undesired immune reactions for the bacteria, thus jeopardising colonisation. Furthermore, the immunogenic property of FH binding protein is demonstrated by being a main component of two meningococcal Serogroup B vaccines (extensively reviewed by Seib, KL *et al* [32]). We previously demonstrated that pneumococcal PspC is predominantly located and exposed at the division septum [33]. This suggests that the surface located PspC could trigger a reaction of the adaptive immune system. In addition, fine tuning and quick adaption of PspC expression via an RNAT, allows the bacteria to remain undetected by immune effectors, as long as low environmental temperature persists.

**Fig 5:**
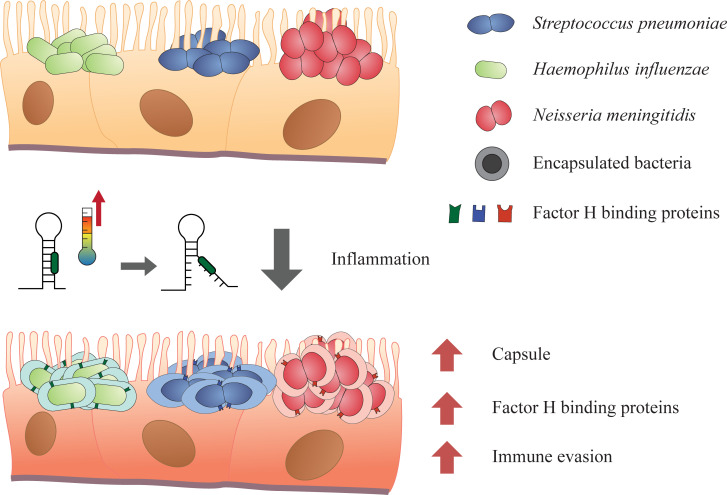
Model of pathogenesis, from commensalism to systemic infection. Mucosal surface inflammation (e.g. by trauma or infection) raises the temperature, leading to increased expression of virulence determinants such as polysaccharide capsules and FH binding proteins of *S*. *pneumoniae*, *H*. *influenzae* and *N*. *meningitidis*. This thermoregulation enables the bacteria to evade immune responses on the mucosal surfaces. The bacteria are primed to higher temperature and might have a better chance of surviving within the body, possibly increasing the rate of systemic infections. Green box in RNA structure denotes ribosome binding site. Model not according to scale.

Our work using assays for serum killing and opsonophagocytosis demonstrates that both *S*. *pneumoniae* and *H*. *influenzae* are able to evade complement-mediated killing at higher temperature. An increase in temperature triggered by inflammation in the host may act as a ‘danger signal’ for *S*. *pneumoniae* and *H*. *influenzae* priming their defences against the recruitment of immune effectors onto the mucosal surface. While attuned to higher threat, it remains largely unknown how these pathogens breach from the mucosa into the bloodstream and further into the brain. We hypothesise that the nasopharyngeal tissue could be damaged during prior influenza A viral infections, serving as an entry site for the bacteria. Temperature increment caused by a local inflammation by primed pathogens could enhance evasion of immune responses and increase bacterial growth, leading to bacteraemia and the pathogen crossing the blood brain barrier to cause meningitis [34–36]. A proposed infection model influenced by temperature is shown in Fig 5.

In conclusion, we show that the human restricted respiratory pathogens, *S*. *pneumoniae* and *H*. *influenzae*, sense ambient temperature changes. RNATs positively influence the expression of their major virulence determinants at warmer growth conditions. Interestingly, the four novel RNATs described here, together with the three known neisserial RNATs [14], do not possess any sequence similarity, but all retain the same thermosensing ability. Our observation here suggests that while nucleotide sequences within the 5´-UTR could be dispensable, a functional RNAT imperatively depends on its secondary structure with a weakly base-paired RBS. It is most likely that these RNATs in *S*. *pneumoniae*, *H*. *influenzae* and *N*. *meningitidis* have evolved independently to sense the same temperature cue in the nasopharyngeal niche to avoid immune killing. Temperature plays a major role in bacterial fitness, whether the phenotypical differences could be attributed to the RNAT alone or if other factors were involved remains to be investigated.

RNA-mediated virulence gene regulation in bacterial pathogens is still an understudied phenomenon. The pathogenesis of *S*. *pneumoniae* and *H*. *influenzae*, and their dual nature in harmless colonisation or initiator of lethal infection makes the carrier state equally important for investigation, especially on the role of these RNATs. With recent RNA sequencing methods such as RNA structurome sequencing analysis [37], comprehensive RNAT maps could be generated. In addition, nuclear magnetic resonance (NMR) spectroscopy could be used to investigate the RNA structures [38–40]. Our work here demonstrates the involvement of RNATs in immune evasion of *S*. *pneumoniae* and *H*. *influenzae*. Future studies encompassing *in vivo* infection model are warranted to characterise the role of these RNATs during colonisation and invasion. In this study, due to low availability of nBLAST hits from *S*. *pneumoniae* and *H*. *influenzae* isolates (Table 1), we were not able to investigate and assert stronger role of these RNATs with invasive disease. Extensive RNAT allele designation could benefit *S*. *pneumoniae* and *H*. *influenzae* pathogenesis research, exemplified by our work in *Neisseria meningitidis* [16]. Discoveries of RNATs, as well as other regulatory RNAs, in clinical isolates could identify markers for monitoring of hypervirulent strains and improve understanding of their roles in bacterial pathogenesis as well as in invasive disease progression.

## Materials and methods

### Bacterial strains, plasmids and growth conditions

Strains, oligonucleotides and plasmids are listed in Table 2. *Streptococcus pneumoniae* were grown on blood agar plates, and *Haemophilus influenzae* were grown on GC II Agar with Haemoglobin and IsoVitaleX at 30°C—42°C and 5% CO_2_ overnight. *E*. *coli* was grown in Luria–Bertani (LB) agar at 37°C overnight. When necessary, antibiotic was added to the following final concentration: kanamycin, 50μg/mL. Liquid cultures of *S*. *pneumoniae* were grown in C + Y media at 32°C– 40°C, taken from 20% glycerol stocks of a *S*. *pneumoniae*-culture grown to mid-logarithmic phase at the same temperature. Liquid cultures of *H*. *influenzae* were started from a *H*. *influenzae* culture grown on plate over-night at 37°C and 5% CO_2_. The new culture was incubated at 32°C in 25mL supplemented BHI (BHI broth + 0.1% Hemin, 0.1% NAD+) (sBHI) in 250mL-Erlenmeyer flasks and shaking at ~180rpm. Upon reaching OD_600_ = 0.5 (mid-logarithmic phase) the culture was diluted 1:10 in 25mL sBHI pre-warmed to 32°C—40°C and grown at the intended temperature in 250μL-Erlenmeyer flasks and ~180rpm.

**Table 2:** Oligonucleotides, strains and plasmids.

Primers	Sequence (5´-3´)	Restriction Enzymes
CspA(GFP)-F	GGGGGAATTCTTCTAAAACATTGTTAGAAATCG	*EcoRI*
CspA(8C-GFP)-R	GGGGCCCGGGTGATTTTTTAAAACGTCTACTCAT	*XmaI*
BscA(GFP)-F	GGGGGAATTCTTAACACCGCACAGACCGC	*EcoRI*
BscA(8C-GFP)-R	GGGGCCCGGGCAACACCACCAGCTAGAATG	*XmaI*
CssA(GFP)-F	GGGGGAATTCCCAAATTCAAGATTTGCGAGG	*EcoRI*
CssA(8C-GFP)-F	GGGGCCCGGGTGTAATGCAAAGAATTCTTTTCAT	*XmaI*
PspC(GFP)-F	GGGGGAATTCAGAGATAAATACAAAATTCGATTTA	*EcoRI*
PspC(30C-GFP)-R	GGGGCCCGGGAACAAGACTGGCAACAACTACAC	*XmaI*
PH(GFP)-F	GGGGGAATTCATGAAACGGCTAATTCAAGATG	*EcoRI*
PH(30C-GFP)-R	GGGGCCCGGGTTGAGTATTATCACCGCCGC	*XmaI*
CspA(qPCR)-F	CTTAATCTAGTGGTAACTGCG	
CspA(qPCR)-R	CCAACAAACTGCTGTACTGC	
Bsc(qPCR)-F	GCTGGAGCATACTCTTGCC	
Bsc(qPCR)-R	TTCTGGTTCATCTTGAAGAGC	
CssA-U	ACCAGAGCCGATTCGGC	
CssA-D	GTTCAATTTCATCAGATAGACG	
pspC(qPCR)-F	CCCACTTCTTCTAATAGGGC	
pspC(qPCR)-R	ATCATCAGTATCTGTAGTTGGC	
PH(qPCR)-F	ACGCTTGCCAACTAATACCG	
PH(qPCR)-R	CTTGCAATTGCACCAAAGCG	
fHbp-U	AAACGAGAAACTGAAGCTGGC	
fHbp-D	CACTCTCCAAGGTAATGAGC	
tmRNA(TIGR4)-F	CGTTACGGATTCGACAGGC	
tmRNA(TIGR4)-R	ACAAGGCTTAATCGTATCTCG	
tmRNA(HI)-F	CGAAGCCCAAGGTGCACG	
tmRNA(HI)-R	AGTGCTTCGATCCTCAAACG	
**Strains and plasmids**		**Reference**
*Streptococcus pneumoniae* serotype 4 strain TIGR4 (T4)		[41], ATCC BAA-334
*Streptococcus pneumoniae* T4R		[42]
*Streptococcus pneumoniae* serotype 1 clinical isolate		BHN31[43]
*Streptococcus pneumoniae* serotype 2 clinical isolate		BHN147, from the BHN collection
*Streptococcus pneumoniae* serotype 22F clinical isolate		BHN794, from the BHN collection
*Streptococcus pneumoniae* serotype 2 strain D39		[44]
*Streptococcus pneumoniae* strain IU1781 (D39 *rpsL1*)		[45]
*Streptococcus pneumoniae* serotype 19F strain NUH0014		Clinical isolate
*Streptococcus pneumoniae* strain NUS0114 (D39 *rpsL1* Δ*cps*::P-*sacB*-*kan*-*rpsL*^+^)		This study
*Streptococcus pneumoniae* strain NUS0276 (D39 *rpsL1 cps19F*)		This study
*Haemophilus influenzae* serotype B (10810)		NCBI Reference Sequence: NC_016809.1
*Escherichia coli* XL10-Gold		Agilent Technologies
pEGFP-N2 plasmid		[14]

In order to generate the CpsA-GFP, BscA-GFP, CssA-GFP, PspC-GFP and PH-GFP constructs in the plasmid pEGFP-N2 [14], the following oligonucleotides were used to amply the fragments from their respective gDNAs; CspA(GFP)-F with CpsA(8C-GFP)-R, BscA(GFP)-F with BscA(8C-GFP)-R, CssA(GFP)-F with CssA(8C-GFP)-R, PspC(GFP)-F with PspC(30C-GFP)-R and PH(GFP)-F with PH(30C-GFP)-R. PCR fragments together with pEGFP-N2 plasmid were then digested with *EcoRI* and *XmaI* restriction enzymes (New England Biolab) according to manufacturer’s protocol and ligated using T4 ligase (Promega). The resulting constructs were sequenced to ensure that no other changes had occurred prior transformation into XL10-Gold *E*.*coli* (Agilent).

Strain NUS0114 was constructed by transforming genomic DNA of strain SpnYL001 (a generous gift from the laboratory of Marc Lipsitch) into strain IU1781 (D39 rpsL1). Transformation was performed as described by inducing natural competence in *S*. *pneumoniae* [46–48]. The resulting strain NUS0114 (D39 *rpsL1 Δcps*::*P-sacB-kan-rpsL*^*+*^) was selected on blood agar plates supplemented with kanamycin (250 μg/ml) and transformed with the genomic DNA of strain NUH0014. Transformants were selected for sucrose (9% (w/v)) and streptomycin (300 μg/ml) resistant as well as smooth colony morphology, followed by PCR verification [49]. Genomic DNA was purified from the resulting strain and transformed again into strain NUS0014 to remove unwanted genetic elements from the clinical isolate other than the serotype 19F capsule locus [50]. The production of serotype 19F capsule in the final strain, NUS0276, was confirmed by Quellung reaction using antiserum against serotype 19F (SSI) and validated by PCR.

### RNA isolation and northern blotting

*S*. *pneumoniae*, and *H*. *influenzae* were grown in liquid culture to an OD_600 nm_ of ~0.5 (5x10^8^ CFUs/mL; 2.5x10^9^ CFUs/mL;) before RNA extraction. RNA was isolated using the FastRNA Pro Blue Kit (MP Biomedical) following the manufacturer’s protocol. Northern blot analyses were performed according to standard protocols. Briefly, PCR probes were generated using oligonucleotide pairs CpsA(qPCR)-F with CpsA(qPCR)-R, Bsc(qPCR)-F with Bsc(qPCR)-R, CssA-U with CssA-D, pspC (qPCR)-F with pspC(qPCR)-R, PH(qPCR)-F with PH(qPCR)-R, fhbp-U with fhbp-D and tmRNA(TIGR4)-F with tmRNA(TIGR4)-R, tmRNA(HI)-F with tmRNA(HI)-R. PCR fragments were then labelled using Megaprime DNA Labeling Systems (GE Healthcare). Primers used are listed in Table 2.

### *In vitro* transcription/translation

Plasmids containing the desired constructs were *in vitro* transcribed in an *E*. *coli* S30 Extract system for Linear Templates kit (Promega) according to the manufacturer’s instructions. In brief, *cpsA-gfp*, *bscA-gfp*, *cssA-gfp*, *pspC-gfp*, *lph-gfp* and *prfA-gfp* plasmids (for plasmid constructs see bacterial strains, plasmids and growth conditions) were digested using NotI restriction enzyme and purified using QiAquick PCR purification kit (Qiagen). One microgram of *cpsA-gfp*, *bscA-gfp*, *cssA-gfp*, *pspC-gfp*, *lph-gfp* and *prfA-gfp* digested plasmids mixtures were incubated at 30°C, 32°C, 34°C, 36°C, 38°C and 40°C for 1h before transferring onto ice for 5 min. Samples were acetone-precipitated, re-suspended in 1X sample buffer, and separated on a 12% polyacrylamide gel before being transferred onto Trans-Blot Turbo Midi (PVDF) membranes (Biorad) using the Trans-Blot Turbo Transfer System (Biorad). Membranes were developed following the protocol of the ECL western blotting kit (Amersham), using anti-GFP (BD-living colours) as primary antibody and an HRP-conjugated anti-mouse as the secondary antibody (GE Healthcare).

### SDS–PAGE and western blotting

*S*. *pneumoniae*, and *H*. *influenzae* were grown in liquid culture to an OD_600_ of ~0.5 before protein isolation. Total protein was isolated from whole cell lysates using 0.1% Triton at 37°C for 10min (*S*. *pneumoniae*) or 1% SDS at 50°C for 30min (*H*. *influenzae*). Total protein concentrations were quantified by Pierce BCA quantification kit (Thermo Fisher Scientific). 20μg of protein was loaded into each lane of the gel. Western blot analyses were performed according to standard protocols. For western blot analysis, membranes were washed three times in 0.05% (w/v) dry milk/PBS with 0.05% (v/v) Tween-20 for 10 min, and then incubated with the primary antibody for 1 h. Membranes were washed again three times and incubated for a further hour with a secondary, horseradish peroxidase (HRP)-conjugated antibody for 1 h. Binding was detected with an ECL Western Blotting Detection kit (Amersham) and exposed to ECL Hyperfilm. Anti-CpsB rabbit antibody (gift from Professor James C. Paton) was used at a dilution of 1:1000. Anti-CbpA/PspC rabbit antibody (gift from Professor Elaine I. Tuomanen) was used at a dilution of 1:8000. Anti-Lph rabbit antibody (gift from Professor Kristian Riesbeck) was used at a dilution of 1:5000. Anti- fHbp Anti-RecA rabbit antibody (Abcam) was used at a dilution of 1:8000. Anti-LytA rabbit antibody was used at a dilution of 1:2000. Anti-GAPDH rabbit antibody was used at a dilution of 1:2000. Anti-fHbp mouse antibody was used at a dilution of 1:5000. Anti-GFP mouse antibody (BD living colors) was used at a dilution of 1:8000. Anti-Neomycin Phosphotransferase (Npt) rabbit antibody (Novus) was used at a dilution of 1:1000. For Far western, Complement factor H from human plasma (Sigma Aldrich) was used at a dilution of 1:10000 prior to Anti-Factor H goat antibody at a dilution of 1:1000. HRP-conjugated anti-mouse, anti-rabbit or anti-goat as the secondary antibody (GE Healthcare) were used at a dilution of 1:3000.

### Serum killing assay

To determine the effect of temperature on complement sensitivity, *H*. *influenzae* was grown in liquid media according to our previous published protocol [14]. In brief, *H*. *influenzae* strain was grown on chocolate agar plates overnight at 30°C and re-suspended in PBS. Bacteria were diluted to a final concentration of 1 × 10^7^ c.f.u. ml^−1^ in RPMI medium (Gibco). To compare the sensitivity of bacteria at different temperature, resuspended bacteria in RPMI were then split and incubated at 30°C and 37°C for a further 1 h. One-million c.f.u. were incubated with 25% human immune serum (Sigma-Aldrich) for 30 min, and the proportion of bacteria surviving was determined by plating 10 μl aliquots onto chocolate plates and counting the number of colonies after overnight incubation; differences were analysed with the Student’s *t*-test.

### Opsonophagocytosis assay

*S*. *pneumoniae* was grown on blood agar plates at 30°C or 37°C and 5% CO_2_ overnight. Colonies were inoculated into C+Y medium and grown until exponential phase (~5x10^8^ CFUs/mL) at 30°C or 37°C prior to incubation with THP-1 macrophages. Serial dilutions were plated on blood agar plates and incubated over night at 37°C and 5% CO_2_ to determine the number of phagocytosed bacteria.

### Polysaccharide dot blot

*S*. *pneumoniae* and *H*. *influenzae* were grown in liquid as described above. Bacteria were grown at 32°C, 37°C, or 40°C until cultures reached OD_600_ = 0.5. Cells and medium were split by centrifugation and supernatant isolated. *H*. *influenzae* supernatant was digested with Proteinase K (Qiagen) in 30mM Tris-HCL pH8, 10mM EDTA, 1% SDS at 50°C for 1h.1:10 dilutions of the supernatants were blotted on a supported nitrocellulose membrane (Amersham Protran Supported NC Nitrocellulose, Cytiva of Danaher Corp.) using vacuum and the Bio-Dot Microfiltration Apparatus (Bio-Rad), and subsequently blocked in 5% milk. Capsule components of *S*. *pneumoniae* TIGR4 were detected using 1:2 optimised rabbit type-4 antiserum (Statens Serum Institute) and 1:20,000 anti-Rabbit-HRP. Optimised antiserum was produced by exposing live T4R (non-encapsulated TIGR4) to Type 4 antiserum, 1:100 diluted in PBS for 30 min at 37°C. Capsule components of *H*. *influenzae* were detected using 1:5000 diluted rabbit anti-serotype-B antiserum (Statens Serum Institute) and 1:20,000 anti-Rabbit HRP.

### Flow cytometry analysis and fluorescence microscopy

Bacteria were grown in liquid from -80°C 20% glycerol stocks, prepared from cultures in mid-logarithmic phase. *S*. *pneumoniae* grew in C + Y media, and H. *influenzae* in BHI media at 30°C, 32°C, 37°C, or 40°C until cultures reached 5x10^8^ (*S*. *pneumoniae*) or 2.5x10^9^ (*H*. *influenzae*) CFU/mL. Binding of human FH (Merck Millipore) was detected with Alexa-Fluor-488 labelled human FH. Labelling was performed with the Alexa Fluor 488 Microscale Protein Labelling Kit. Sample data was acquired on Gallios flow cytometer (Beckman Coulter) and at least 2x10^5^ events recorded. Fluorescence intensity was analysed using Kaluza software (Beckman Coulter). For fluorescence microscopy 5μL of the samples were dried on cover slip, then covered with Vectashield H-1400 and analysed using the DeltaVision Elite (GE Healthcare).

### FITC-dextran exclusion assay

Measurement of the capsule thickness with the exclusion zone of FITC-dextran (FD2000S, Sigma) was performed as described before [50]. Briefly, cells of strain NUS0276 (D39 *rpsL1 cps19F*) were grown overnight on blood agar plates at 30, 37, or 42°C and resuspended in 10 μl of 1x PBS (137 mM NaCl, 2.7 mM KCl, 1 mM CaCl_2_, 0.5 mM MgCl_2_, 10 mM Na_2_HPO_4_, 1.8 mM KH_2_PO_4_, pH 7.4). The suspension was mixed with 2 μl of 10 mg/ml of FITC-dextran and 1 μl of the mixture was mounted on a pre-cleaned glass slide. Bacteria were visualised on an Olympus IX81 platform. Capsule thickness was measured and quantitated using the cellSens software (Olympus).

## Supporting information

S1 Fig 5′-UTR structural analysis of *S. pneumoniae* and *H. influenzae* capsular operon mRNAs and experimental controls. (A) Promoter sequence of the pneumococcal *cpsA* gene (including 5´-UTR, ribosomal binding site (RBS) and coding sequence (CDS). A putative *cpsA* RNAT secondary structure is shown below. (B) Promoter sequence of of the *H*. *influenzae* type b *bcs1´* gene (including 5´-UTR, ribosomal binding site (RBS) and coding sequence (CDS). A putative *bcs1´* RNAT secondary structure is shown below. (C) Western blot of positive control of 5´-UTR-*gfp* fusion products in *E*. *coli*. *cssA* (*N*. *meningitidis*) or *prfA* (*L*. *monocytogenes*). The respective 5´-UTRs were fused with *gfp* and expressed from a plasmid in *E*. *coli* grown at different temperature. (Anti-GFP antibody used for detection of GFP, RecA and Neomycin-phosphotransferase antibodies used as loading controls). (D) Western blots of *in vitro* transcription/translation assays of positive controls CssA and PrfA UTR-gfp fusion products show temperature regulation of CssA-GFP and PrfA-GFP. (Anti-GFP antibody used for detection of GFP).
(TIF)

S2 Fig Northern Blots show no change in mRNA transcript levels (capsule and factor H binding protein) with increasing temperature. *cpsA* and *bcs1’* are the first genes of the capsular operons. *pspC* and *lph* are genes for the factor H binding in *S*. *pneumoniae* TIGR4 and *H*. *influenzae* type b proteins. Transfer messenger RNA (*tmRNA*) was used as control.
(TIF)

S3 Fig 5′-UTR structural analysis of *S. pneumoniae* and *H. influenzae* factor H binding protein mRNAs, experimental controls and flow cytometry analysis. (A) Promoter sequence of the pneumococcal *pspC* gene (including 5´-UTR, ribosomal binding site (RBS) and coding sequence (CDS). Putative *pspC* RNAT secondary structure is shown on the right. (B) Promoter sequence of the *H*. *influenzae* type b *lph* gene (including 5´-UTR, ribosomal binding site (RBS) and coding sequence (CDS). Putative *lph* RNAT secondary structure is shown on the right. (C) Western blot of positive controls, 5´-UTR-gfp fusion products FHbp (*N*. *meningitidis*) and PrfA (*L*. *monocytogenes*) in an *in vitro* translation / transcription assays. (Anti-GFP antibody used for detection of GFP). (D) Fluorescent flow cytometry shows increased human FH binding to *S*. *pneumoniae* and *H*. *influenzae* type b (Hib) when grown at higher temperature. In *H*. *influenzae* a subpopulation can be seen to increasingly bind FH binding when grown at 40°C.
(TIF)

S4 Fig Sequence comparison of the *cpsA* 5´-UTR in different pneumococcal isolates. Polymorphisms (red) indicated within the putative secondary structures of the *S*. *pneumoniae cpsA* mRNA (see S1A Fig).
(TIF)

S5 Fig Sequence comparison of the *pspC* 5´-UTR in different pneumococcal isolates. Polymorphisms (red) indicated within the putative secondary structures of the *S*. *pneumoniae pspC* mRNA (see S3A Fig).
(TIF)
