# Supplementary figures and images for: RNA thermosensors facilitate Streptococcus pneumoniae and Haemophilus influenzae immune evasion
Source: PLoS Pathog. 2021 Apr 29;17(4):e1009513. doi: 10.1371/journal.ppat.1009513 (PMC8084184; doi:10.1371/journal.ppat.1009513)

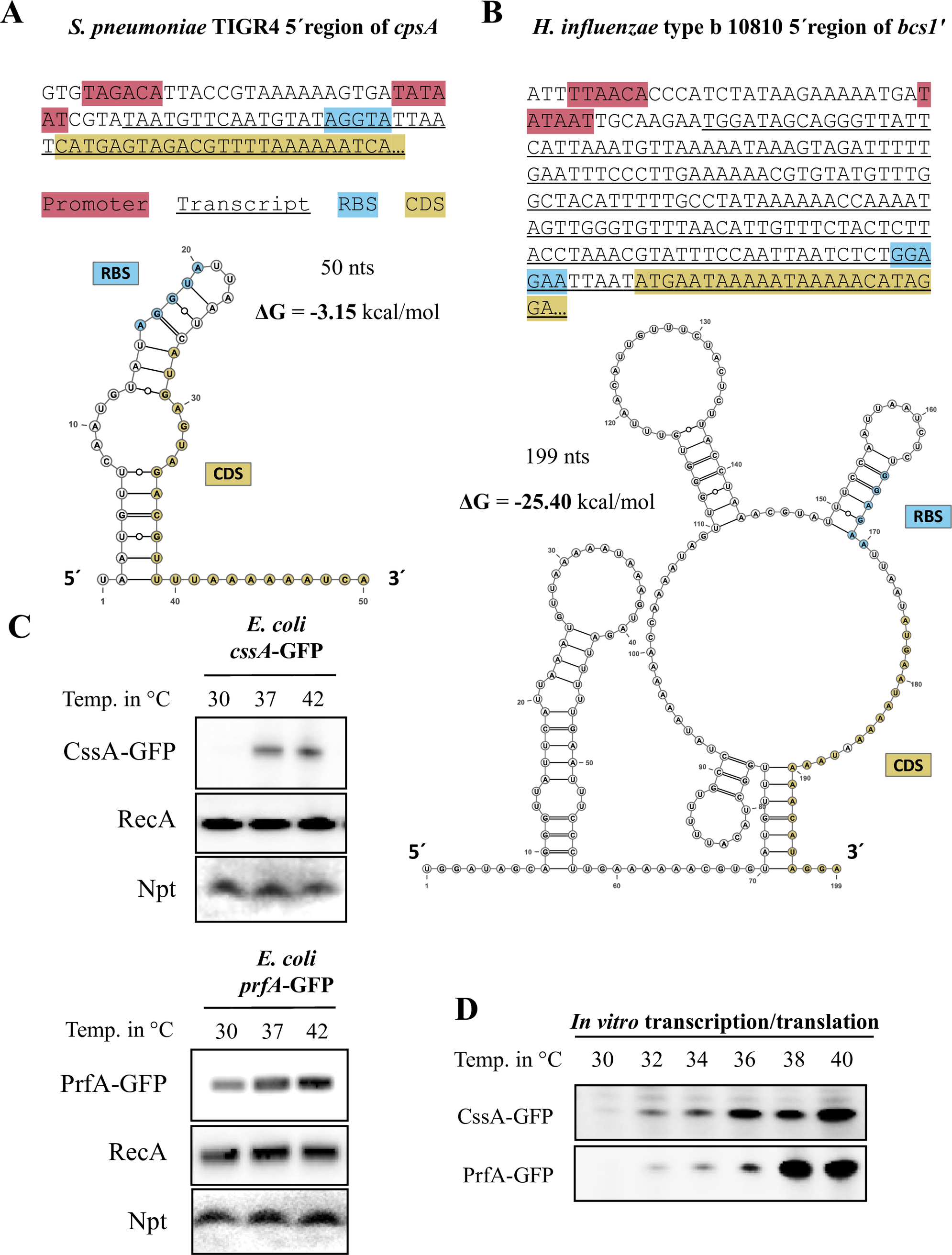

Supplement: S1 Fig — (A) Promoter sequence of the pneumococcal cpsA gene (including 5´-UTR, ribosomal binding site (RBS) and coding sequence (CDS). A putative cpsA RNAT secondary structure is shown below. (B) Promoter sequence of of the H. influenzae type b bcs1´ gene (including 5´-UTR, ribosomal binding site (RBS) and coding sequence (CDS). A putative bcs1´ RNAT secondary structure is shown below. (C) Western blot of positive control of 5´-UTR-gfp fusion products in E. coli. cssA (N. meningitidis) or prfA (L. monocytogenes). The respective 5´-UTRs were fused with gfp and expressed from a plasmid in E. coli grown at different temperature. (Anti-GFP antibody used for detection of GFP, RecA and Neomycin-phosphotransferase antibodies used as loading controls). (D) Western blots of in vitro transcription/translation assays of positive controls CssA and PrfA UTR-gfp fusion products show temperature regulation of CssA-GFP and PrfA-GFP. (Anti-GFP antibody used for detection of GFP). (TIF) [file ppat.1009513.s001.tif]

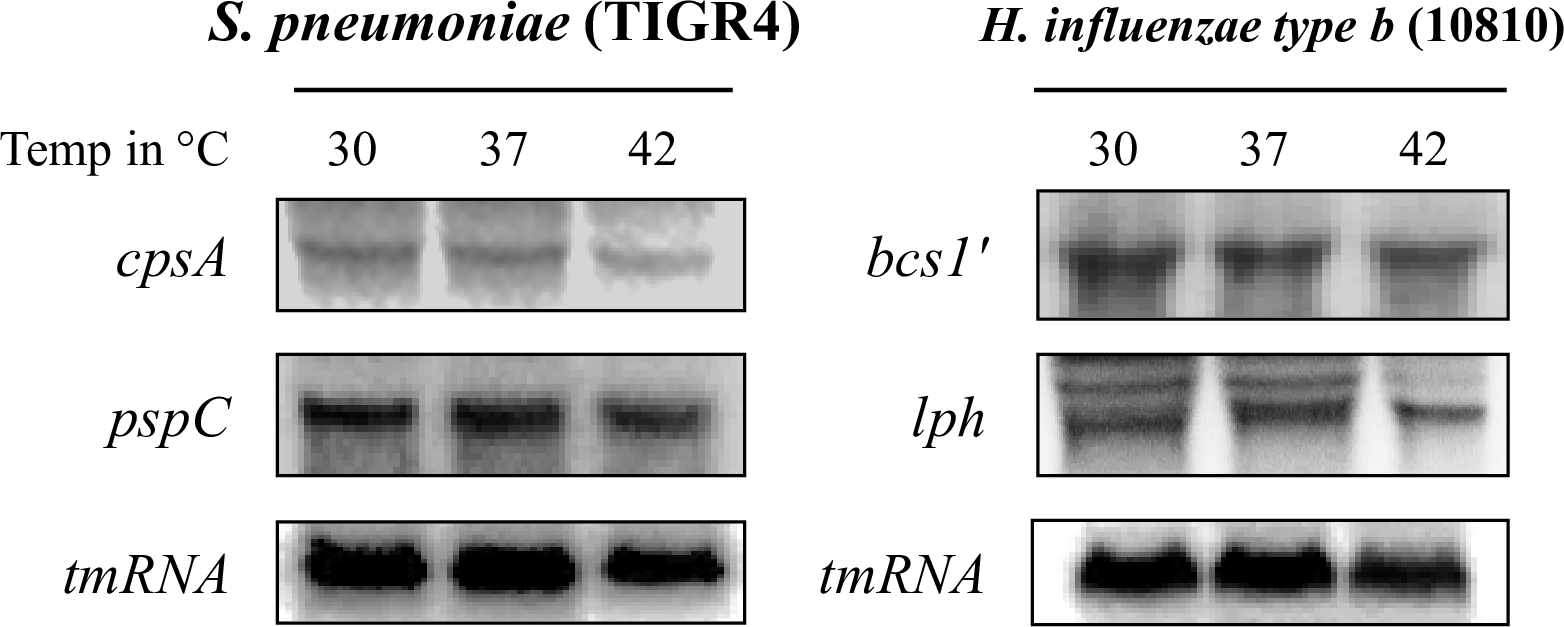

Supplement: S2 Fig — cpsA and bcs1’ are the first genes of the capsular operons. pspC and lph are genes for the factor H binding in S. pneumoniae TIGR4 and H. influenzae type b proteins. Transfer messenger RNA (tmRNA) was used as control. (TIF) [file ppat.1009513.s002.tif]

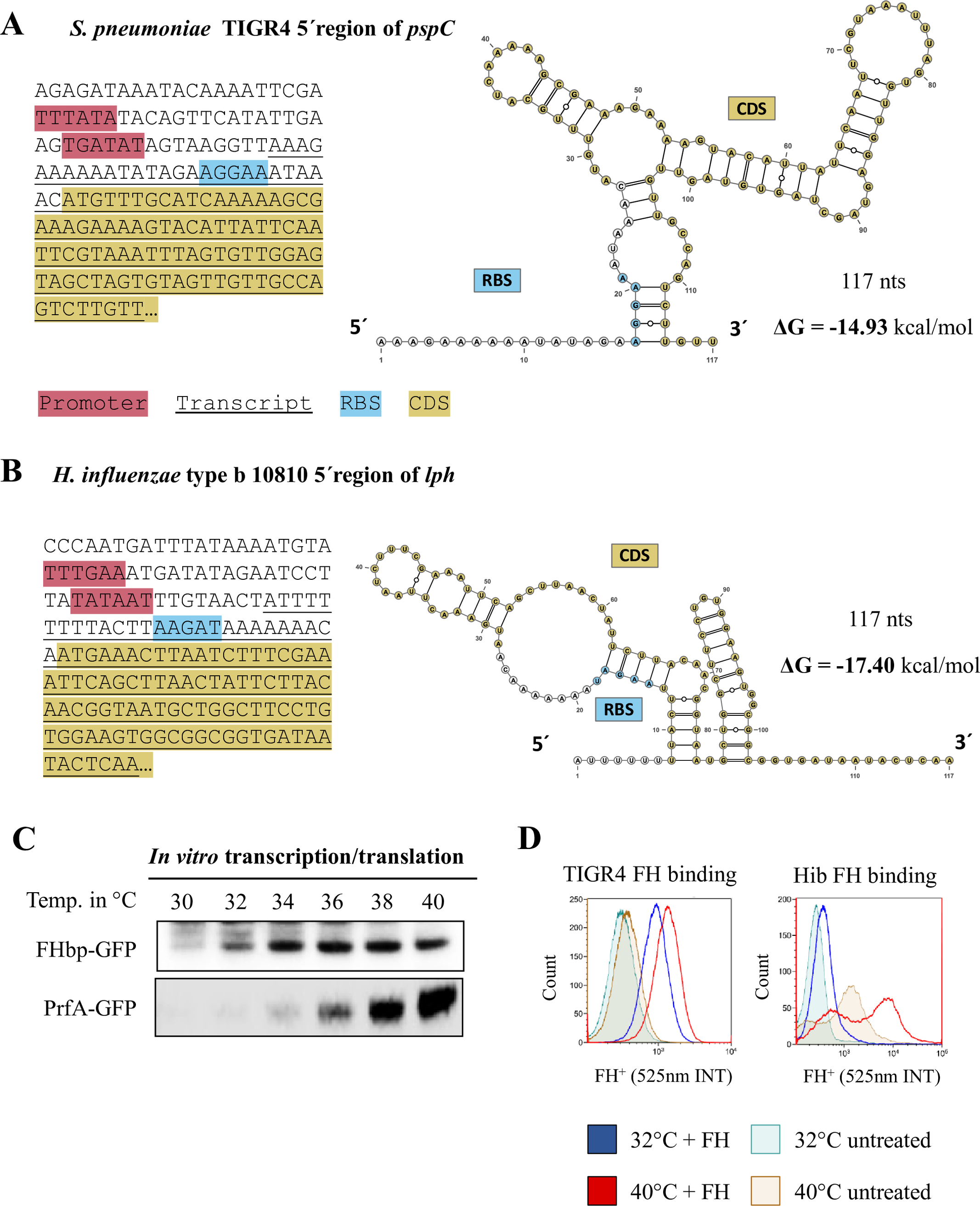

Supplement: S3 Fig — (A) Promoter sequence of the pneumococcal pspC gene (including 5´-UTR, ribosomal binding site (RBS) and coding sequence (CDS). Putative pspC RNAT secondary structure is shown on the right. (B) Promoter sequence of the H. influenzae type b lph gene (including 5´-UTR, ribosomal binding site (RBS) and coding sequence (CDS). Putative lph RNAT secondary structure is shown on the right. (C) Western blot of positive controls, 5´-UTR-gfp fusion products FHbp (N. meningitidis) and PrfA (L. monocytogenes) in an in vitro translation / transcription assays. (Anti-GFP antibody used for detection of GFP). (D) Fluorescent flow cytometry shows increased human FH binding to S. pneumoniae and H. influenzae type b (Hib) when grown at higher temperature. In H. influenzae a subpopulation can be seen to increasingly bind FH binding when grown at 40°C. (TIF) [file ppat.1009513.s003.tif]

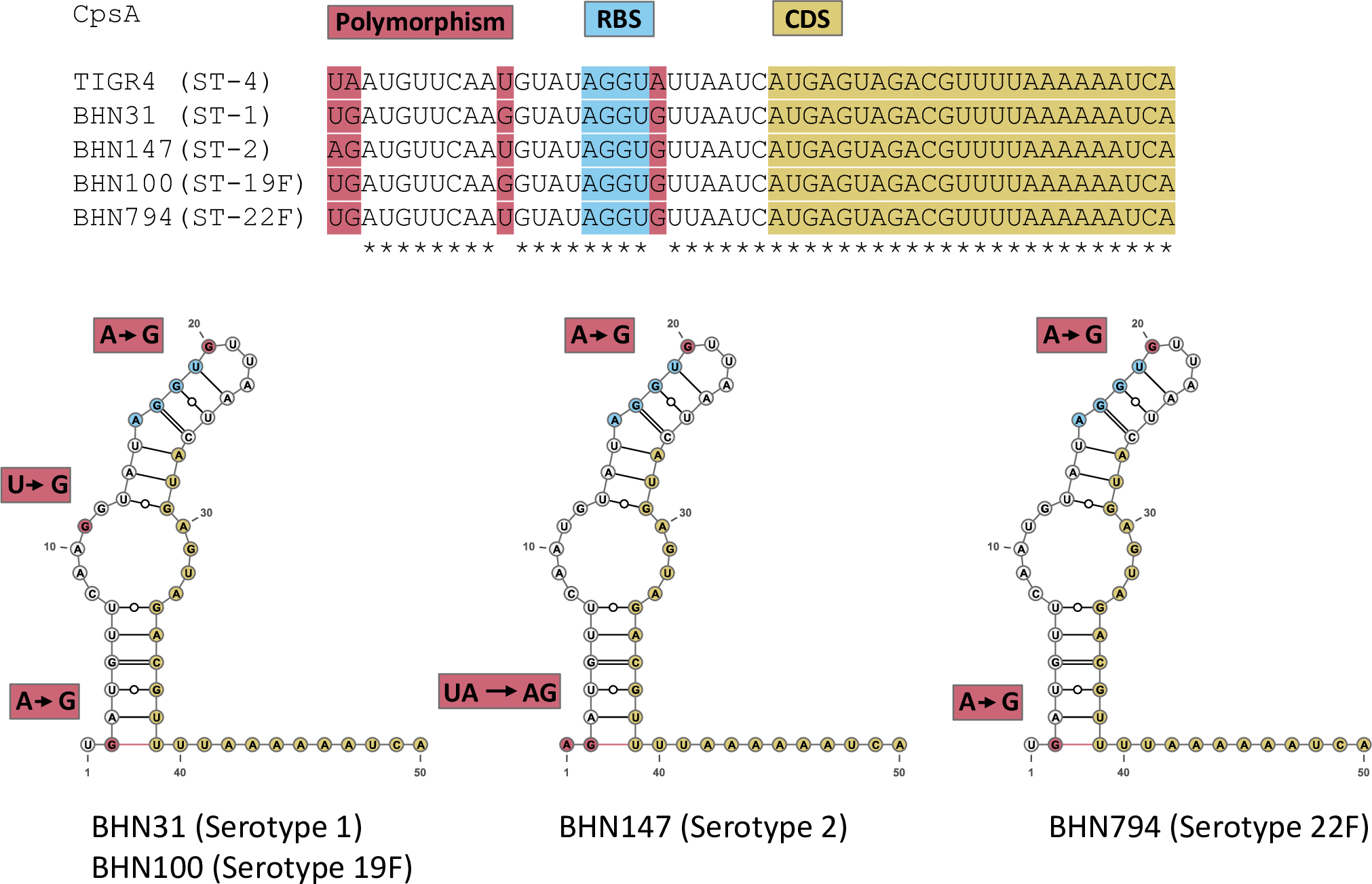

Supplement: S4 Fig — Polymorphisms (red) indicated within the putative secondary structures of the S. pneumoniae cpsA mRNA (see S1A Fig). (TIF) [file ppat.1009513.s004.tif]

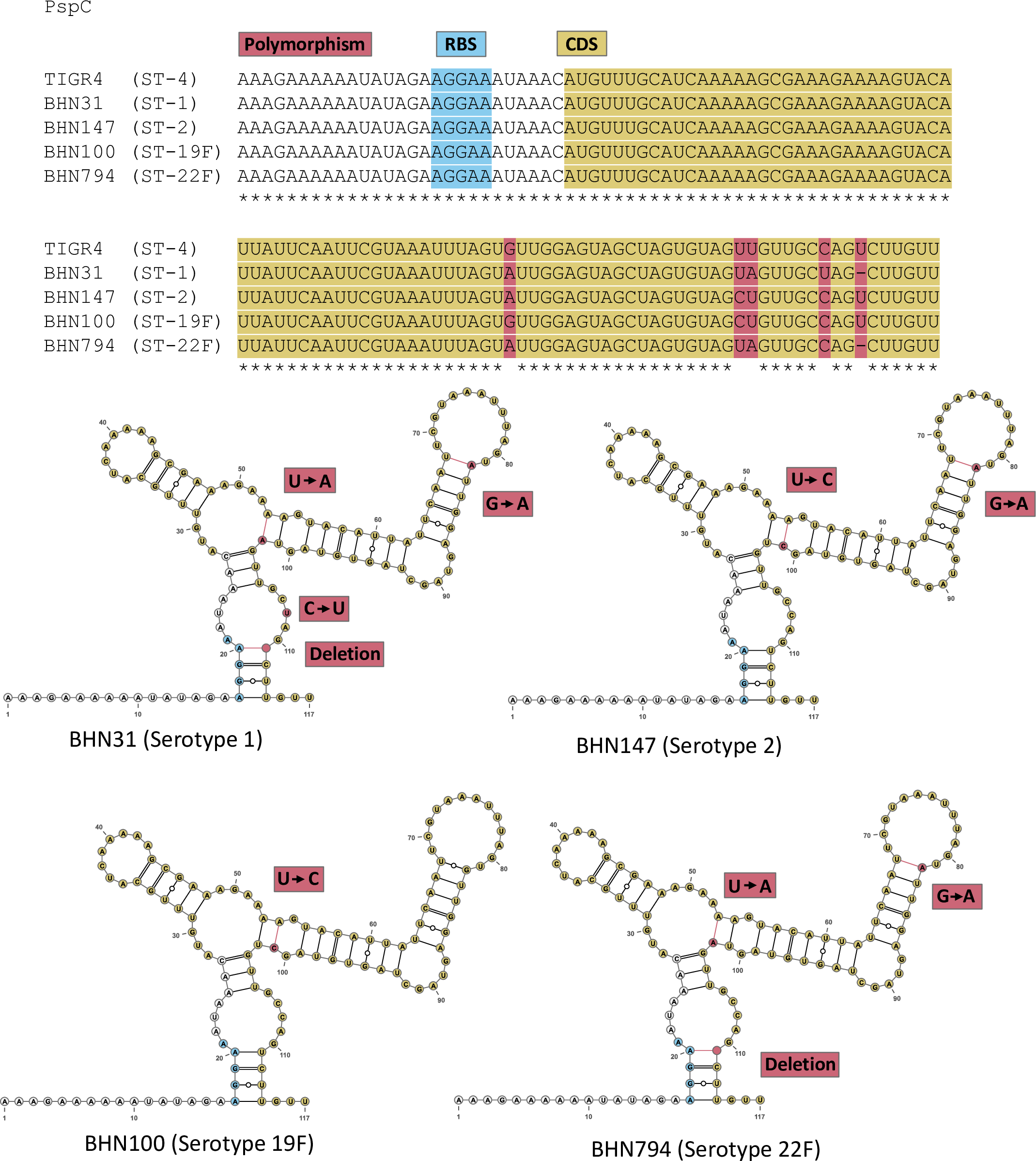

Supplement: S5 Fig — Polymorphisms (red) indicated within the putative secondary structures of the S. pneumoniae pspC mRNA (see S3A Fig). (TIF) [file ppat.1009513.s005.tif]
